# Arabic translation, cross-cultural adaptation, feasibility, acceptability, and initial psychometric evaluation of the hot flushes diary and the hot flush rating scale and diary interference scale (HFRDIS) in young breast cancer survivors

**DOI:** 10.1186/s41687-026-01103-3

**Published:** 2026-06-03

**Authors:** Maram Montaser, Fatma Abdalkarim Ibrahim, Fatma Bektash, Hayam Ateyya, Nouran Omar El Said, Emad Shash

**Affiliations:** 1 Patient Education Unit, Breast Cancer Comprehensive Center, National Cancer Institute, Cairo University, Giza, Egypt; 2 Department of Pharmacy Practice & Clinical Pharmacy, Faculty of Pharmacy, Future University in Egypt, Cairo, Egypt; 3 Medical Oncology Department, National Cancer Institute, Cairo University, Giza, Egypt

## Abstract

**Background:**

Hot flushes (HF) are a highly prevalent and distressing vasomotor symptom among young breast cancer survivors receiving endocrine therapy, yet validated Arabic patient-reported instruments are limited for quantifying daily symptoms and their interference with functioning.

**Methods:**

The 3-Category Hot Flushes Diary and the Hot Flash Related Daily Interference Scale (HFRDIS) were translated using forward–back translation and culturally adapted into Arabic. A total of 37 premenopausal women aged ≤ 45 years with breast cancer receiving adjuvant endocrine therapy were prospectively enrolled at the National Cancer Institute, Cairo University. Participants completed the Arabic HFRDIS once (reflecting the preceding week). Then they completed the Arabic diary for seven consecutive days, recording daily hot-flash frequency and maximum daily severity. Internal consistency was assessed using Cronbach’s α (acceptability ≥0.70). Descriptive statistics summarized symptom frequency/severity and HFRDIS interference scores. Feasibility was assessed using completion rate and participant feedback on clarity and cultural appropriateness.

**Results:**

Participants had a mean age of 38.5 ± 4.7 years; most were receiving tamoxifen (94.6%) and goserelin (73.0%). The Arabic diary demonstrated excellent internal consistency (Cronbach’s α = 0.933), with stable item-deletion α values (0.924–0.933). Median daily HF frequency was 2 episodes/day (range 0–9), with mean daily frequency ranging from 2.2 to 2.6 episodes/day across the week. Moderate-to-severe symptoms were frequently observed. The Arabic version of the HFRDIS exhibited high reliability, with a Cronbach’s alpha of 0.908. and indicated the greatest interference in mood (mean 8.0), sleep (mean 7.5), and concentration (mean 6.9), with notable interference in work, leisure activities, and overall quality of life. The sexuality item had fewer responses than other domains. All participants judged the instruments clear and culturally appropriate, and completion of the diary was 100%.

**Conclusions:**

The Arabic 3-Category Hot Flushes Diary and Arabic HFRDIS demonstrated strong internal consistency and feasibility in this initial evaluation and appear suitable for use in Egyptian populations. These tools may support standardized symptom assessment and clinical decision-making in similar settings. Future studies should evaluate test–retest reliability and responsiveness in larger and more diverse Arabic-speaking populations.

**Supplementary Information:**

The online version contains supplementary material available at 10.1186/s41687-026-01103-3.

## Introduction

Hot flashes (HF) are sudden, short-lasting episodes of intense warmth, commonly accompanied by visible flushing, profuse sweating, palpitations, and a sense of anxiety or discomfort. Physiologically, these episodes are marked by peripheral vasodilation, which leads to increased cutaneous blood flow and elevated skin temperature across multiple regions, including the face, chest, arms, back, abdomen, legs, and digits [1–4]. 

HF are among the strongest predictors of reduced quality of life, with greater symptom severity linked to lower physical, psychological, and social functioning. Beyond symptom distress, HF may contribute to disrupted sleep, fatigue, reduced work productivity, and impaired adherence to long-term therapies, ultimately compromising daily activities and overall well-being, highlighting the need for accurate assessment and management [5]. 

A recent global systematic review and meta-analysis, including 321 studies and 482,067 women, found that HF is one of the most prevalent vasomotor symptoms worldwide, with a pooled global prevalence of about 52.7%. Rates varied by region, reaching 64.4% in Africa, and were higher in low-income countries (≈ 65.9%) compared with high-income settings (≈ 49.7%). These findings highlight that HF affects more than half of middle-aged women globally, emphasizing their significant health impact and the importance of reliable assessment tools across cultures and languages [6].

Over the past several years, HF has come to be recognized as a significant source of morbidity in women treated for breast cancer and in healthy women using tamoxifen for prevention. Recognizing this issue, the North Central Cancer Treatment Group (NCCTG) initiated efforts in 1989 to design clinical trials aimed at evaluating potential treatments for HF in breast cancer survivors, as well as in women who avoided estrogen therapy due to concerns about breast cancer risk. When Sloan et al. initiated their first hot flash study in 1989, no validated instrument existed to measure HF adequately in clinical trial settings [7]. 

A well-established psychometric method involves asking patients to document their perceived frequency and intensity of clear, observable symptoms in a diary. For this reason, Sloan et al. chose to use patient diaries to capture subjective assessments of hot flash activity. They developed a structured hot-flash diary with four severity categories, using simple, concrete language and a practical, common-sense structure. This instrument collected daily information from patients on the total number of HF episodes experienced, as well as ratings of each episode’s severity—categorized as mild, moderate, severe, or very severe [7]. The descriptions for each of the four hot flash severity categories are provided on a separate form, maintained alongside the primary data collection form, to enhance clarity and reduce potential confusion during the recording of patient-reported data [8]. 

After completing an initial hot flash clinical trial that employed the 4-category diary [9], Guttuso et al. observed that approximately half of the participants, according to the Principal Investigator’s recollection, reported confusion regarding the severity definitions provided in the diary. This observation led Guttuso et al. to develop and implement a 3-category diary that aligned with FDA and EMEA guidelines [8].

Although objective methods are available to quantify the frequency of hot flashes, there remains no gold standard for evaluating their subjective impact on daily functioning and overall quality of life. To address this gap, the Hot Flash Related Daily Interference Scale (HFRDIS) was developed as a targeted instrument that specifically assesses how HF interfere with nine distinct daily activities, work, social activities, leisure activities, sleep, mood, concentration, relationships with others, sexuality, and enjoyment of life (items 1–9) as well as overall quality of life (item 10). This focused framework provides a more precise and clinically meaningful evaluation of the burden that HF imposes on patients’ everyday lives [10]. 

Given the high prevalence and substantial impact of HF on women’s daily functioning and quality of life, particularly in the Middle East, there is a growing need for research that supports the development of effective management strategies. Such efforts require valid and reliable tools to measure both clinical improvement and the daily burden of hot flashes.

The objective of this study was to translate the 3-category hot flash diary, and the Hot Flash Related Daily Interference Scale (HFRDIS) into Arabic and to conduct their validation, an initial psychometric evaluation assessing internal consistency and feasibility.

This was primarily done to enable their use in a larger parent study monitoring hot flashes in young breast cancer patients, ensuring the instruments are culturally appropriate and suitable for both research and clinical practice.

## Methods

### Ethical approval

Ethical approval for the parent study protocol was obtained from the Institutional Review Board (IRB) of the National Cancer Institute (Approval Number: MO2501-105-096-195) and the Research Ethics Committee of Future University in Egypt (Approval Number: REC-FPFUE-15/2025). The study was prospectively registered at ClinicalTrials.gov before the enrollment of participants (Registration Number: NCT06774885). All study procedures adhered to the principles of the Declaration of Helsinki, and written informed consent was obtained from all participants.

### Study design

This study performed Arabic linguistic validation (translation and cultural adaptation) and initial psychometric evaluation of two patient-reported instruments used to assess hot flashes: the 3-Category Hot Flushes Diary and the Hot Flash Related Daily Interference Scale (HFRDIS). Before initiating Arabic linguistic validation, cultural adaptation, feasibility assessment, and initial psychometric evaluation of the 3-Category Hot Flushes Diary and the Hot Flash Related Daily Interference Scale (HFRDIS), the authors contacted the original developers/owners of these instruments and obtained approval to conduct the translation and validation process. The original instruments and their related publications were appropriately cited throughout the manuscript.

The psychometric assessment focused on internal consistency and feasibility. Structural validity, test–retest reliability, responsiveness, and measurement invariance were not evaluated in this preliminary phase.

The instruments were translated into Arabic using a standard forward–backward translation process. Two independent translators performed the forward translation, followed by reconciliation into a single version. A back-translation was then conducted to ensure conceptual equivalence. Cognitive debriefing interviews were carried out with a sample of patients to assess clarity, comprehensibility, and cultural appropriateness. Feedback from these interviews was incorporated into the final Arabic version.

The validation aimed to ensure conceptual equivalence, clarity, and cultural appropriateness of the Arabic versions, followed by field testing to assess feasibility and internal consistency. In the field phase, participants completed the HFRDIS once to reflect interference during the preceding week and then completed the hot-flash diary prospectively for seven consecutive days to capture daily hot-flash frequency and maximum severity.

### Translation and cultural adaptation

Both instruments were translated into Arabic and culturally adapted to ensure semantic and conceptual equivalence with the original versions. Independent forward translations were produced and reconciled into a single harmonized Arabic version, with careful attention to maintaining the intended meaning of key symptom descriptors and response options while using clear, patient-friendly language. The reconciled Arabic version was then back-translated into English to verify semantic equivalence with the source instruments, and any inconsistencies or ambiguities were discussed and resolved by consensus within the study team. An expert review by clinicians experienced in breast cancer survivorship and symptom assessment further ensured medical accuracy, cultural suitability, and clarity across all items and instructions. The translated versions were reviewed with participants from the target population during the initial in-person administration to confirm clarity, comprehension, and cultural appropriateness. Participants were encouraged to indicate any unclear wording or interpretation difficulties before beginning the recording phase. No major comprehension problems were identified, and only minor linguistic refinements were required. The finalized Arabic versions were then used for the evaluation in the study sample (*n* = 37).

### Study setting

The study was conducted at the Breast Cancer Comprehensive Center, National Cancer Institute (NCI), Cairo University, which serves women from diverse Egyptian geographical and cultural backgrounds. Potential participants were identified by daily screening of clinic files, and all interviews and study procedures were conducted in private consultation rooms to ensure confidentiality and participant comfort.

### Participants

Eligible participants were female breast cancer patients aged 45 years or younger who were premenopausal at diagnosis and receiving endocrine therapy for at least three months prior to recruitment. Inclusion criteria comprised female sex, age ≥ 18 years, a confirmed diagnosis of breast cancer, current treatment with adjuvant endocrine therapy, and experience of bothersome hot flashes occurring at least 14 times per week. These criteria were adopted from the original parent study to maintain consistency with its design.

The age cutoff of ≤ 45 years was selected based on epidemiological and survivorship literature indicating that breast cancer diagnosed at or before this age is commonly classified as young-onset disease, a group characterized by distinct clinical features, increased likelihood of treatment-induced menopausal symptoms, and specific psychosocial needs. Previous studies and reviews have used similar thresholds (≤ 40–45 years) to define young breast cancer survivors, supporting the appropriateness of this cutoff for survivorship research [11–14]. Participants had to be able to understand the interview questions and complete a seven-day diary. Women were excluded if they were older than 45 years, were not receiving endocrine therapy at recruitment, did not experience hot flashes at the time of recruitment, had cognitive impairment that interfered with study procedures, or were unable to complete the diary due to illiteracy or reading/writing difficulties.

### Procedures and instruments

After eligibility confirmation and consent, each participant completed the Arabic HFRDIS in a private interview. The HFRDIS uses a 0–10 numeric rating scale, with higher scores indicating greater interference, and participants were instructed to rate the extent of interference across the HFRDIS domains based on their experience during the preceding week. Immediately after the interview, participants received the Arabic Hot Flushes Diary in paper format and were instructed to complete it for seven consecutive days. Each day, participants recorded the total number of hot flashes experienced (frequency) and selected one option indicating the maximum severity experienced that day using the provided definitions (no hot flash, mild, moderate, or severe).

### Follow-up and data collection

During the seven-day recording period, participants could be contacted by phone or WhatsApp to provide supportive reminders, clarify previously explained instructions, and respond to participant questions regarding diary completion. All instructions and severity definitions were standardized and explained in person at enrollment before diary initiation. Follow-up contacts did not include reinterpretation of symptoms, prompting responses, or guidance that could influence reporting, and were intended solely to support adherence and accurate completion of the diary.

### Sample size

The present analysis represents the validation component of a larger prospective study. The overall study sample size was calculated as a priori using G*Power (version 3.1.9.2), assuming a medium effect size of 0.3, α = 0.05, and 80% power, resulting in a minimum of 111 participants (37 per group). To compensate for potential dropout, the target sample was increased to 120 participants [15–18].

The evaluation reported here was conducted in participants who completed the diary phase (*n* = 37), representing an initial feasibility and reliability assessment of the translated instruments.

### Statistical analysis

Statistical analyses were performed using IBM SPSS^®^ Statistics version 26 (IBM^®^ Corp., Armonk, NY, USA). Descriptive statistics were used to summarize participant characteristics and responses to both instruments; continuous variables were reported as mean (standard deviation) or median (range), as appropriate, and categorical variables were reported as counts and percentages. Internal consistency reliability of the Arabic Hot Flushes Diary and Arabic HFRDIS were evaluated using Cronbach’s alpha (α). Cronbach’s alpha was calculated by treating the seven daily diary entries as parallel measurement occasions contributing to a 7-day composite symptom-burden score rather than as independent content items. The aim was to provide an initial estimate of the reliability of aggregating daily recordings across the week and to assess whether participants applied the standardized severity definitions consistently throughout the recording period. This analysis was therefore interpreted as an indicator of temporal consistency in symptom reporting rather than internal consistency of a multi-item scale. Response distributions and patterns of missing data were examined to inform the initial assessment of feasibility and measurement performance in the target population. Formal construct or criterion validity analyses were not performed in this preliminary validation phase. For the HFRDIS, domain-level descriptive statistics were calculated using available responses. Overall HFRDIS reliability estimates were calculated among participants with complete item-level data, consistent with the item–total (item-deletion) statistics reported.

## Results

A total of 37 women were included, representing a predominantly young and married population undergoing endocrine therapy (Table 1). The mean age was 38.5 years (median 39). Most participants were receiving tamoxifen-based therapy (94.6%), and nearly three-quarters were receiving ovarian suppression with goserelin (73.0%). Targeted therapy exposure was uncommon (10.8%), and the duration of endocrine therapy ranged from less than one year to seven years (Table 1).

Table 1. Sociodemographic and clinical characteristics of the study participants (n = 37), including age, marital status, endocrine treatment type, use of ovarian suppression, targeted therapy exposure, and duration of endocrine therapy. *Percentages may not total 100 due to rounding.*

**Table 1:** Sociodemographic and treatment characteristics of the study participants (*n* = 37)

Domain	Category	N	%
Marital status	Single	3	8.1
	Married	28	75.7
	Divorced	5	13.5
	Widowed	1	2.7
Endocrine therapy	Tamoxifen	35	94.6
	Aromatase Inhibitors	2	5.4
Ovarian suppression (goserelin)	No	10	27.0
	Yes	27	73.0
Targeted therapy exposure	No	33	89.2
	Yes	4	10.8
Duration of endocrine therapy (years)	< 1	10	27.0
	1	3	8.1
	2	9	24.3
	3	6	16.2
	4	4	10.8
	5	2	5.4
	6	1	2.7
	7	2	5.4

### Arabic hot flushes diary performance and symptom profile

Internal consistency of the Arabic Hot Flushes Diary was excellent. Cronbach’s alpha for the diary severity component was 0.933, and alpha values remained stable with minimal variation across item-deletion statistics (Table 2), supporting the homogeneity of the diary construct across the recording period. For the frequency component, internal consistency was similarly high, with Cronbach’s alpha values consistently around 0.924–0.927 across days (Table 2).

**Fig. 1:**
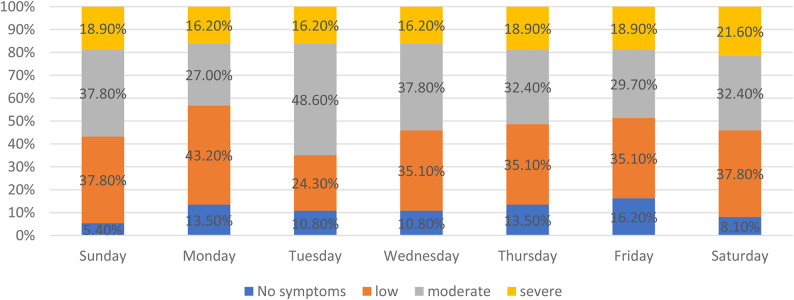
Day-by-day distribution of hot flush severity recorded using the arabic hot flushes diary (7-Day Period)

Across the seven-day recording period, participants reported hot flashes of varying severity on most days. Moderate-to-severe symptoms were observed throughout the week, with the proportion reporting moderate symptoms ranging from 27.0% to 48.6% and severe symptoms ranging from 16.2% to 18.9% across the recorded days (Fig. 1). The proportion reporting no symptoms varied between 5.4% and 13.5% depending on the day (Fig. 1). Daily hot-flash frequency was relatively stable over time, with a median of two episodes per day on all recorded days and mean daily frequency ranging from 2.2 to 2.6 episodes/day (Table 3).

**Table 2:** Internal consistency reliability of the arabic hot flushes diary across the 7-day recording period

Day	Cronbach’s α (Severity)	Cronbach’s α (Frequency)
Sunday	0.933	0.927
Monday	0.930	0.924
Tuesday	0.931	0.924
Wednesday	0.932	0.925
Thursday	0.929	0.926
Friday	0.929	0.925
Saturday	0.932	0.926
Overall	**0.933**	**—**

Table 2 presents internal consistency reliability of the Arabic Hot Flushes Diary severity and frequency components across the seven consecutive recording days, expressed as Cronbach’s alpha (α). The overall Cronbach’s alpha for the severity component was 0.933, indicating excellent internal consistency.

Figure 1 illustrates the distribution of daily maximum hot flush severity recorded over seven consecutive days using the Arabic Hot Flushes Diary. Severity is categorized as no hot flush, mild, moderate, or severe, based on standardized definitions provided to participants. The figure highlights day-to-day patterns in symptom severity across the observation period and summarizes the proportion of participants reporting each severity category per day.

**Table 3:** Daily hot flush frequency recorded over 7 consecutive days using the arabic hot flushes diary (*n* = 37)

Day	*n*	Mean	SD	Median	Min	Max
Sunday	37	2.4	1.6	2.0	0	7
Monday	37	2.2	1.8	2.0	0	8
Tuesday	37	2.3	1.6	2.0	0	6
Wednesday	37	2.3	1.9	2.0	0	9
Thursday	37	2.5	1.8	2.0	0	6
Friday	37	2.6	2.1	2.0	0	8
Saturday	37	2.6	1.9	2.0	0	9

Table 3 summarizes daily hot flush frequency (episodes/day) recorded prospectively over seven consecutive days using the Arabic Hot Flushes Diary. Values are presented as mean (SD), median, and range (minimum–maximum) for each day.

### Arabic HFRDIS performance and interference profile

**Fig. 2:**
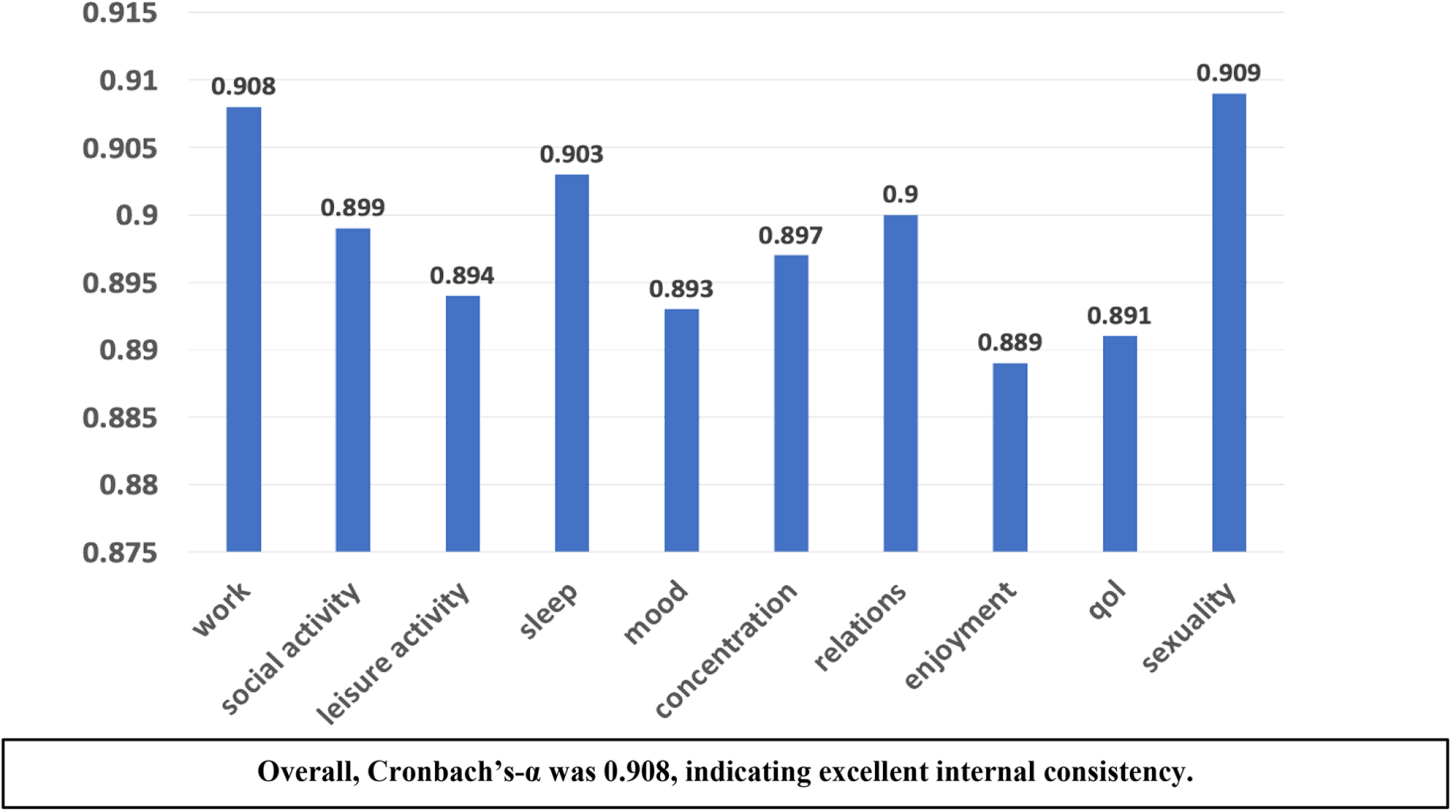
Internal consistency reliability of the arabic hot flash related daily interference scale (HFRDIS)

The Arabic HFRDIS demonstrated excellent internal consistency, with Cronbach’s alpha of 0.908 (Fig. 2). Domain-level descriptive statistics showed that hot flashes interfered most with mood (mean 8.0, median 9), sleep (mean 7.5, median 9), and concentration (mean 6.9, median 8), indicating a substantial psychosocial and cognitive burden (Table 4). Interference was also notable in leisure activities (mean 6.4, median 8), overall quality of life (mean 6.4, median 8), and work (mean 5.9, median 6) (Table 4).

The sexuality domain demonstrated greater variability and had a smaller valid sample (*n* = 32) compared with other items (*n* = 37), reflecting item non-response or “not applicable” responses. However, the overall reliability remained strong, and the scale showed stable performance across domains (Fig. 2).

Figure 2 illustrates the internal consistency reliability of the Arabic version of the Hot Flash Related Daily Interference Scale (HFRDIS), assessed using Cronbach’s alpha (α) for each domain based on item–total statistics. All domains demonstrated strong internal consistency. The sexuality domain had fewer valid responses (*n* = 32) because five participants selected “not applicable,” whereas all other domains had complete responses (*n* = 37).

**Table 4:** Hot flash–related interference with daily functioning measured by the arabic HFRDIS (0–10 Scale)

HFRDIS domain	*N*	Mean	SD	Median	Min	Max
Work	37	5.9	3.1	6.0	0	10
Social activities	37	5.6	3.5	7.0	0	10
Leisure activities	37	6.4	3.3	8.0	0	10
Sleep	37	7.5	3.1	9.0	0	10
Mood	37	8.0	2.7	9.0	0	10
Concentration	37	6.9	3.5	8.0	0	10
Relationships with others	37	5.6	4.1	7.0	0	10
Sexuality*	32	4.9	4.6	6.0	0	10
Enjoyment of life	37	5.4	3.7	6.0	0	10
Overall quality of life	37	6.4	3.1	8.0	0	10

Table 4 summarizes domain-level interference scores measured by the Arabic Hot Flash Related Daily Interference Scale (HFRDIS). Each domain is rated on a 0–10 numeric scale (0 = no interference; 10 = complete interference). Values are presented as mean (SD), median, and range (minimum–maximum). *The sexuality domain has fewer valid responses (*n* = 32) due to “not applicable” or missing responses, while all other domains include all participants (*n* = 37).

## Discussion

The purpose of this study was to translate and perform Arabic linguistic validation and initial psychometric evaluation of the Arabic versions of the 3-Category Hot Flashes Diary and the Hot Flash Related Daily Interference Scale (HFRDIS). The instruments demonstrated excellent internal consistency (α > 0.90); these high values indicate strong coherence among items within each scale, consistent with the underlying theoretical constructs rather than item redundancy and support the reliability of the Arabic versions among Egyptian women enrolled at the study center.

These findings indicate that the translated tools were reliably understood by Egyptian women and performed in a manner consistent with internationally recognized standards for cross-cultural adaptation, including systematic forward and backward translation, expert panel review, cognitive debriefing, and repeated revisions to make sure the scale was clear, meaningful, and culturally appropriate [19–23]. However, linguistic and cultural differences across Arabic-speaking populations may influence responses, particularly for sensitive items such as sexuality-related content. Therefore, caution is warranted when generalizing these findings, and further cross-cultural validation is recommended.

The strong reliability of the Arabic diary aligns with broader methodological evidence demonstrating that well-designed prospective symptom diaries can produce stable and valid data across diverse populations [24, 25]. Prospective documentation is particularly valuable in hot flash research, as it reduces recall bias and enhances ecological validity, advantages consistently supported in prior diary-based studies [24–27]. These results support the utility of the Arabic diary as a precise tool for capturing real-time day-to-day variability in vasomotor symptoms.

The findings of this study demonstrate that the Arabic HFRDIS not only reliably measures the multidimensional impact of hot flashes on daily functioning, mood, sleep, and overall quality of life, but also captures patterns of interference that are consistent with the original scale and prior research [28–30]. Hot flashes had the greatest impact on mood, sleep, and concentration, with moderate effects on work, leisure, and overall quality of life, highlighting the domains most vulnerable to vasomotor symptoms in young breast cancer survivors [31–33]. These results reinforce the psychometric robustness of the Arabic adaptation and underscore its practical value for both clinical monitoring and research applications. Importantly, the sensitivity of the scale to meaningful changes in interference suggests it is suitable for tracking symptom progression or evaluating interventions, positioning it as a critical tool for both survivorship care and future studies in Arabic-speaking populations [34, 35]. “Notably, the magnitude of interference scores observed in mood, sleep, and concentration falls within ranges reported in previous studies using the original HFRDIS, supporting their clinical significance and indicating a substantial impact of vasomotor symptoms on daily functioning.”

Cultural considerations were particularly relevant for items related to intimacy and interpersonal relationships, which are often challenging to adapt in conservative cultural contexts [36]. However, the inclusion of the sexuality item did not reduce the scale’s reliability in the Arabic version (α remained between 0.905 and 0.908). This suggests that the adaptation process preserved the intended meaning of the item while ensuring acceptability to participants, aligning recommendations for culturally sensitive translation [20].

## Study strengths

A major strength of this study is its rigorous cross-cultural adaptation process combined with real-world psychometric testing in a clinically relevant population. The inclusion of both symptom frequency/severity (Hot Flushes Diary) and functional impact (HFRDIS) provides a comprehensive assessment framework. The 100% completion rate further supports the feasibility and acceptability of these tools in routine clinical settings [37–39].

## Study limitations

However, several limitations should be acknowledged. This study was conducted at a single center and represents a preliminary validation in a relatively small sample, which was appropriate for an initial assessment of feasibility and internal consistency. However, the limited sample size and single-center design may affect the precision of reliability estimates and restrict the generalizability of the findings. Therefore, the results should be interpreted with caution, and further validation in larger and more diverse populations is warranted.

Second, validation focused primarily on internal consistency; other psychometric properties, such as test–retest reliability and responsiveness to change, were not assessed [37, 40]. Although follow-up contacts were limited to standardized supportive reminders and clarification of instructions, some degree of contact-related reporting influence cannot be completely excluded.

Third, participants who did not experience hot flashes were excluded, as the study focused on evaluating the performance of the instruments among symptomatic women receiving endocrine therapy. While this approach was suitable for assessing severity and interference measures, it limited evaluation across the full symptom range. Additionally, although the sample size (*n* = 37) was sufficient for an initial assessment of internal consistency, larger studies are required to confirm the scale’s structure, responsiveness, and broader applicability. Also, the study was conducted in a single Egyptian center, which may not reflect the cultural and linguistic diversity of wider Arabic-speaking populations, and the specific inclusion criteria may further restrict the generalizability.

Finally, although this study utilized prospective diaries and structured interference scales to capture patients’ experiences, the self-reported nature of these measures should be interpreted in the context of day-to-day symptom variability. As such, some degree of reporting bias or fluctuation in symptom documentation cannot be entirely excluded [26, 41].

## Clinical implications

The validated Arabic Hot Flushes Diary and HFRDIS provide clinicians with reliable, patient-reported tools to systematically assess vasomotor symptoms and their impact on daily life. Their use may facilitate individualized symptom management, guide supportive care interventions, and improve patient–clinician communication, particularly for young breast cancer survivors receiving endocrine therapy. In routine practice, the diary can support brief symptom monitoring over one week, while the HFRDIS provides a structured snapshot of functional interference that can prioritize targeted counseling (e.g., sleep hygiene, mood support) and appropriate non-hormonal symptom management strategies.

## Future research

Future studies should evaluate the responsiveness of these tools to therapeutic interventions and explore their longitudinal relationship with broader quality-of-life measures. Multicenter and multinational studies across Arabic-speaking countries are also warranted to enhance external validity and promote regional standardization of symptom assessment. Further psychometric work should include test–retest reliability, structural validity, and measurement invariance across key subgroups (e.g., different dialect regions, marital status, and literacy-adapted administration), consistent with COSMIN recommendations [24, 26].

## Conclusion

In conclusion, the Arabic versions of the Hot Flushes Diary and the Hot Flash Related Daily Interference Scale are reliable, culturally appropriate, clinically meaningful instruments, and further comprehensive validation studies are warranted.

Their availability supports standardized assessment of hot flashes and their daily-life impact among Egyptian women, including breast cancer survivors receiving endocrine therapy, and provides a foundation for future studies evaluating additional psychometric properties and responsiveness in larger and more diverse populations.

## Supplementary Information

Below is the link to the electronic supplementary material.

Supplementary Material 1

## Data Availability

The dataset(s) supporting the conclusions of this article are available from the corresponding author on reasonable request.
