# Supplementary material for: Arabic translation, cross-cultural adaptation, feasibility, acceptability, and initial psychometric evaluation of the hot flushes diary and the hot flush rating scale and diary interference scale (HFRDIS) in young breast cancer survivors
Source: J Patient Rep Outcomes. 2026 Jun 3;10:94. doi: 10.1186/s41687-026-01103-3 (PMC13241355; doi:10.1186/s41687-026-01103-3)
Supplement: Supplementary file 1 — Supplementary Material 1 [file 41687_2026_1103_MOESM1_ESM.docx]

**Figure 1. Arabic Translation and Cultural Adaptation of the Hot Flash Related Daily Interference Scale (HFRDIS)**


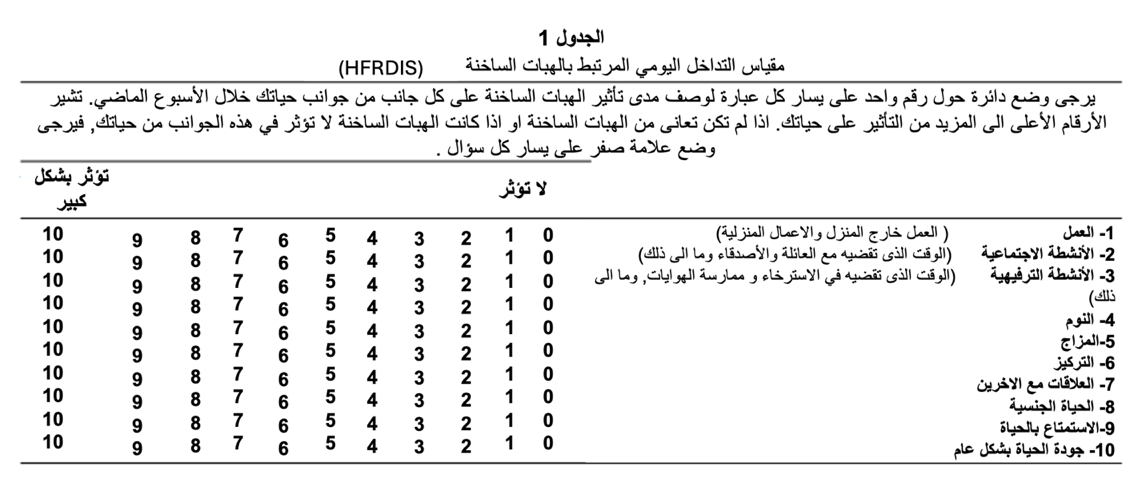


Figure 1 shows the Arabic version of the Hot Flash Related Daily Interference Scale (HFRDIS), adapted from the original study Carpenter J et al. The HFRDIS evaluates the extent to which hot flashes interfere with daily functioning over the preceding week across ten domains: work, social activities, leisure activities, sleep, mood, concentration, relationships with others, sexuality, enjoyment of life, and overall quality of life. Each domain is rated using a 0–10 numeric rating scale (0 = no interference; 10 = complete interference), with higher scores indicating greater perceived interference and functional burden (1).

**Figure 2. Arabic Translation and Cultural Adaptation of the 3-Category Hot Flushes Diary (Daily Frequency and Severity)**

Figure 2 presents the Arabic version of the 3-Category Hot Flushes Diary, adapted from the original instrument developed by Sloan et al., with modifications by Thomas Guttuso Jr. adapted to ensure conceptual equivalence and cultural appropriateness. The diary provides standardized severity definitions and a structured daily recording format for seven consecutive days. Participants document the total number of hot flush episodes each day (frequency) and select one option indicating the severity experienced that day (no hot flush, mild, moderate, or severe), enabling prospective capture of day-to-day symptom burden (2,3).

**References:**

1. Carpenter JS. The Hot Flash Related Daily Interference Scale: A Tool for Assessing the Impact of Hot Flashes on Quality of Life Following Breast Cancer. Journal of Pain and Symptom Management. 2001.

2. Sloan JA, Loprinzi CL, Novotny PJ, Barton DL, Lavasseur BI, Windschitl H. Methodologic Lessons Learned From Hot Flash Studies. Journal of Clinical Oncology. 2001 Dec 1;19(23):4280–90. doi:10.1200/JCO.2001.19.23.4280

3. Guttuso T, Digrazio WJ, Reddy SY. Review of hot flash diaries. Maturitas. 2012. p. 213–6. doi:10.1016/j.maturitas.2011.12.003 PubMed PMID: 22230663.
